# Enhanced tissue infiltration and bone regeneration through spatiotemporal delivery of bioactive factors from polyelectrolytes modified biomimetic scaffold

**DOI:** 10.1016/j.mtbio.2023.100681

**Published:** 2023-05-24

**Authors:** Xiaojun Zhou, Zunjuan Wang, Tao Li, Zhonglong Liu, Xin Sun, Weizhong Wang, Liang Chen, Chuanglong He

**Affiliations:** a State Key Laboratory for Modification of Chemical Fibers and Polymer Materials, Shanghai Engineering Research Center of Nano-Biomaterials and Regenerative Medicine, College of Biological Science and Medical Engineering, Donghua University, Shanghai, 201620, China; b Shanghai Key Laboratory of Orthopaedic Implants, Department of Orthopaedic Surgery, Shanghai Ninth People's Hospital, Shanghai Jiao Tong University School of Medicine, Shanghai, 200011, China; c Department of Oral & Maxillofacial-Head Neck Oncology, Shanghai Ninth People's Hospital, Shanghai Jiao Tong University School of Medicine, Shanghai, 200011, China; d Department of Joint Surgery, Zhongshan Hospital of Traditional Chinese Medicine Affiliated to Guangzhou University of Traditional Chinese Medicine, Zhongshan, 528400, China

**Keywords:** Biomimetic scaffold, Polyelectrolyte modification, Dual-factor delivery, Tissue infiltration, Bone regeneration

## Abstract

Efficient healing of bone defect is closely associated with the structured and functional characters of tissue engineered scaffolds. However, the development of bone implants with rapid tissue ingrowth and favorable osteoinductive properties remains a challenge. Herein, we fabricated polyelectrolytes modified-biomimetic scaffold with macroporous and nanofibrous structures as well as simultaneous delivery of BMP-2 protein and trace element strontium. The hierarchically structured scaffold incorporated with strontium-substituted hydroxyapatite (SrHA) was coated with polyelectrolyte multilayers of chitosan/gelatin *via* layer-by-layer assembly technique for BMP-2 immobilization, which endowed the composite scaffold with sequential release of BMP-2 and Sr ions. The integration of SrHA improved the mechanical property of composite scaffold, while the polyelectrolytes modification strongly increased the hydrophilicity and protein binding efficiency. In addition, polyelectrolytes modified-scaffold significantly facilitated cell proliferation *in vitro*, as well as enhanced tissue infiltration and new microvascular formation *in vivo*. Furthermore, the dual-factor loaded scaffold significantly enhanced the osteogenic differentiation of bone marrow mesenchymal stem cells. Moreover, both vascularization and new bone formation were significantly increased by the treatment of dual-factor delivery scaffold in the rat calvarial defects model, suggesting a synergistic effect on bone regeneration through spatiotemporal delivery of BMP-2 and Sr ions. Overall, this study demonstrate that the prepared biomimetic scaffold as dual-factor delivery system has great potential for bone regeneration application.

## Graphical abstract

**Figure:**
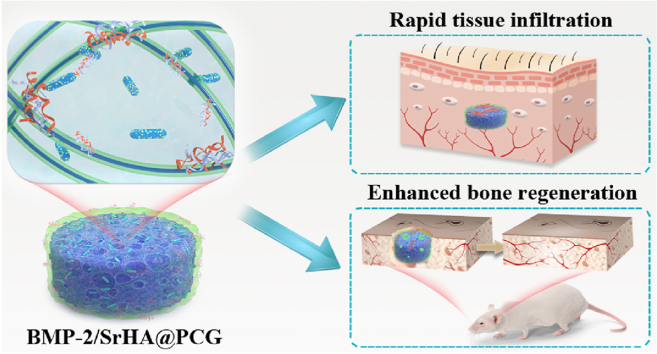

## 1 Introduction

The restoration of bone defects caused by trauma, tumors or bone disease remains a challenge for orthopedic surgeons. Surgical intervention for functional restoration to large bone defects is still frequently required, such as autologous bone grafts and allogeneic bone grafts, which have the advantages of excellent biologic and mechanical properties [1]. However, these traditional approaches are commonly plagued by many problems, including low availability, donor site morbidity, the risk of infection and immune rejection [2]. Alternatively, bone tissue engineering has evolved as a promising strategy for treating bone defects that can overcome these shortcomings. The polymeric biomaterials can be processed into three-dimensional (3D) porous scaffolds with definite shape by various technologies to meet the requirements of bone reconstruction, including providing structural support and guiding bone regeneration after implantation [3]. The biomimetic scaffolds, composed of nanofibrous structure and combination of organic and inorganic components, are widely employed for bone defect healing since they mimic the native bone tissue microenvironment [4,5]. Inspired by the composition of natural bone tissue, the integration of bioactive nanomaterials (e. g. hydroxyapatite, laponite, xonotlite) into polymeric scaffolds exhibits favorable features to induce in-situ bone regeneration, which not only ensures improved mechanical support but also serves as osteogenic stimulators to accelerate bone formation [[6], [7], [8]]. However, the bone regeneration efficacy of organic−inorganic composite scaffolds will be seriously affected when possessed poor tissue ingrowth and osteoinductive properties [[9], [10], [11]]. Thus, the structure and bioactivity of tissue engineered scaffolds should be optimized in order to achieve augmented outcome of bone formation.

To date, electrospinning is always recognized as the most versatile technology to fabricate nanofibrous construct analogous the extracellular matrix (ECM)-like structures [12]. The nanofibrous pattern and diameter play a vital influence on the cell-biomaterial interactions, such as cell adhesion, signal transduction, cell differentiation, and tissue development [13,14]. However, the prepared two-dimensional (2D) membranes cannot meet the requirement of 3D structure for tissue regeneration [15,16]. And it needs complicated process to prepare bulk 3D scaffolds with porous structure by combining other methods, including freeze-drying [17], gas foaming [18], 3D printing [19] and electrohydrodynamic jet writing [20]. Alternatively, thermally induced phase separation (TIPS) was demonstrated to be a facile method to fabricate nanofibrous scaffolds which can accurately mimic ECM structures [21]. To fabricate nanofibrous scaffolds with macroporous architecture by TIPS technology, different porogens are employed to create the porous structure. It is heartening that our previous study had reported to prepare macroporous and nanofibrous scaffold by using blend solution of poly (l-lactic acid) (PLLA)/poly (ε-caprolactone) (PCL) *via* one-pot phase separation technique [22,23]. The PLLA/PCL composite scaffolds had interconnected macropores and nanofibrous structure. Furthermore, with the addition of poly (lactic-*co*-glycolic acid) (PLGA), the pore size and biodegradability of composite scaffold can be controlled [24]. Benefited from the porous architecture, it provided ample space for cell infiltration and also allowed oxygen and nutrients transport throughout the scaffolds [25,26]. However, the composite scaffolds were made from synthetic polymers with weak hydrophilicity which would impair the promotive effect on cell adhesion and proliferation, and rapid tissue regeneration within the scaffolds [27,28]. Besides, as for application in bone tissue engineering, these scaffolds with multilevel microstructure needed further improvement about the osteogenic capacity.

Surface modification has been considered a facile strategy to improve the physicochemical properties and biological functions of tissue engineered scaffolds. Through the surface modification technology, bioactive factors can be immobilized onto scaffolds by various chemical and physical processes to achieve enhanced biological functions compared to the bare one [29]. For example, platelet-derived growth factor-BB (PDGF-BB) was immobilized on the surface of heparin conjugated-PLLA/PLGA/PCL scaffold to enhance the proliferation and migration of vascular smooth muscle cells [24]. Ye and co-workers fabricated the 3D nanofibrous scaffold incorporated with bone morphogenetic proteins-2 (BMP-2)-derived peptides by utilizing a polydopamine (pDA)-assisted coating strategy [30]. Yan et al. reported to prepare a 3D printed scaffold that can control the release of deferoxamine *via* layer-by-layer assembly technique [31]. It was encouraging that the controlled release of bioactive factors from modified scaffolds was realized. After the efficient immobilization of biomolecules, the bioactivity of functionalized scaffolds was accordingly improved. Therefore, the immobilization and controlled release of osteogenic factors on nanofibrous scaffold with multilevel structures suggests the potential ability for rapid cell infiltration and favorable osteogenesis both *in vitro* and *in vivo*.

**Fig. 1:**
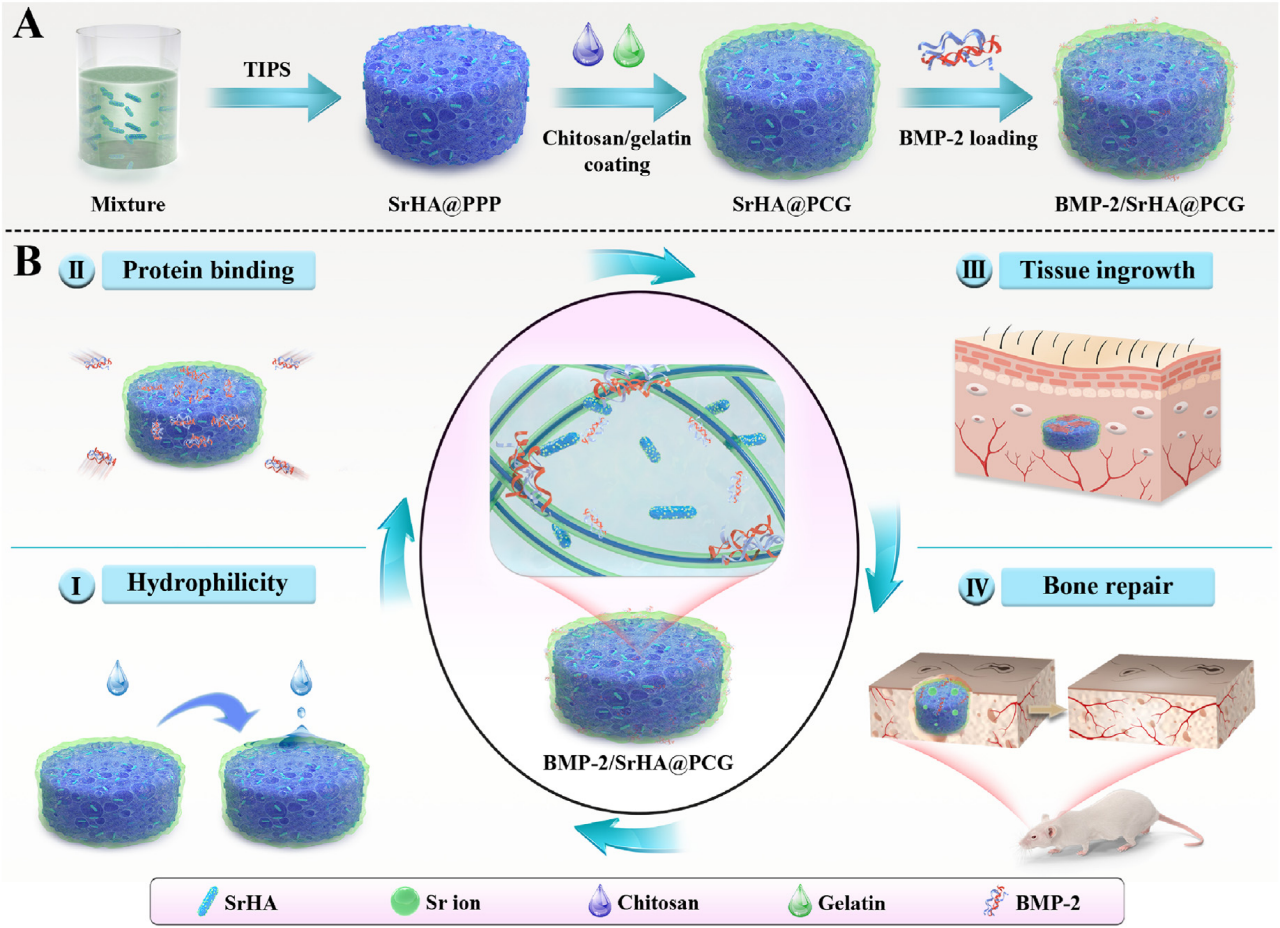
(A) Schematic diagram for the fabrication of BMP-2/SrHA@PCG scaffold. (B) BMP-2/SrHA@PCG scaffold showed excellent hydrophilicity and improved protein binding capacity as well as rapid tissue ingrowth due to the polyelectrolytes modification. With the controlled release of BMP-2 and Sr ions, BMP-2/SrHA@PCG scaffold significantly accelerated the healing of bone defect *via* a synergistically osteogenic response.

Strontium (Sr), as a kind of trace element, is mostly located in bone tissue which plays the pronounced effect in the regulation of bone formation and bone resorption [32]. It was reported that Sr-incorporated scaffolds could promote the *in vitro* proliferation and osteogenic differentiation of bone marrow-derived mesenchymal stem cells (BMSCs) and further enhanced the *in vivo* bone regeneration of bone defects [[33], [34], [35]]. Besides, the Sr-substituted nanomaterials doped into the scaffolds significantly enhanced the mechanical strength [6,8]. As a result, the addition of Sr-substituted nanomaterials into nanofibrous scaffold not only promotes bone regeneration through the osteoinductive effects, but also can increase the mechanical property. Nevertheless, combined delivery of two factors has been demonstrated to exhibit better outcomes in bone regeneration than single factor delivery [[36], [37], [38]]. With these findings in mind, we endeavored to construct a novel dual-factor delivery system based on nanofibrous scaffold with porous structure for accelerating the healing of bone defect (Fig. 1). The strontium-substituted hydroxyapatite (SrHA) was synthesized by hydrothermal treatment and further incorporated into PLLA/PLGA/PCL scaffold with macroporous and nanofibrous structure, followed by the treatment of chitosan/gelatin multilayers for BMP-2 protein immobilization to obtain dual-factor delivery system (denoted as BMP-2/SrHA@PCG scaffold). The incorporation of SrHA could not only improve the mechanical strength of PCG scaffold but also provide a reservoir for Sr ions release. Additionally, the polyelectrolytes modification on scaffolds was intended to increase the hydrophilicity and protein immobilization efficiency, as well as promote cell proliferation and tissue infiltration. Furthermore, BMP-2 and SrHA were orchestrated in the biomimetic scaffold that can allow a rapid release of BMP-2 followed by a long-term sustained release of Sr ions. Accordingly, the promotive effect of BMP-2/SrHA@PCG scaffold on *in vivo* bone regeneration was evaluated in a rat calvarial defects model through micro-CT and histological analysis.

## 2 Materials and methods

### 2.1 Materials

PCL (Mn: 80,000) and gelatin (type B, from bovine skin) were received from Sigma-Aldrich Trading Co., Ltd. (Shanghai, China). PLLA (1.93 ​dL/g inherent viscosity) and PLGA (50:50, 0.46 ​dL/g inherent viscosity) were purchased from Daigang Biomaterials Inc. (Jinan, China). Chitosan (viscosity: 100–200 ​mPa ​s, deacetylation ≥95%) was obtained from Aladdin Industrial Corporation (Shanghai, China). Strontium nitrate (Sr(NO_3_)_2_), calcium dinitrate tetrahydrate (Ca(NO_3_)_2_·4H_2_O), ammonium dihydrogen phosphate (NH_4_H_2_PO_4_) and ammonium hydroxide (NH_4_OH) were purchased from Sinopharm Chemical Reagent Co., Ltd. (Shanghai, China). BMP-2 was acquired from Nanjing GenScript Biotech Co., Ltd. (Nanjing, China). Fluorescein isothiocyanate labelled bovine serum albumin (FITC-BSA) was provided from Beijing Solarbio Science & Technology Co., Ltd. (Beijing, China). Cell Counting Kit-8 (CCK-8), calcein-AM, Alkaline Phosphatase Assay Kit and BCA Protein Assay Kit were obtained from Beyotime Biotechnology Co., Ltd. (Shanghai, China). All chemicals were used as received without further purification.

### 2.2 Fabrication of SrHA-incorporated scaffolds

Sr-containing HA nanoparticles were synthesized by hydrothermal method according to previous report [39]. The molar ratio of Sr/(Ca ​+ ​Sr) was fixed at 10%. The bare PLLA/PLGA/PCL scaffold (denoted as PPP) and SrHA-incorporated PPP scaffold (denoted as SrHA@PPP) were fabricated by the dual phase separation technique [24]. Briefly, PLLA, PLGA and PCL were weighted at weight ratio of 40:30:30 and dissolved in tetrahydrofuran (THF) at 60 ​°C to get homogeneous solutions. The total polymers concentration in THF solvent was fixed at 10% (wt./v). Afterward, the polymers solution was immediately transferred into the molds and phase separated at −80 ​°C overnight. The polymers gel was formed and taken out to subject to the ice/water mixture for exchanging THF. The ice/water mixture was changed three times within 24 ​h. After 48 ​h, the THF-free scaffolds were obtained by freeze-drying [22,24]. Similarly, the SrHA@PPP scaffolds were prepared by mixing of SrHA (10 ​wt% relative to the total polymers) and polymers solution before phase separation process.

### 2.3 Self-assembling of polyelectrolytes on scaffolds

First, 5 ​mg/mL of Chitosan was prepared by dissolving in 1% (v/v) acetic acid. Gelatin was dissolved in phosphate buffered saline (PBS, pH ​= ​7.4) with a final concentration of 5 ​mg/mL. Then the pristine PPP and SrHA@PPP scaffolds with appropriate size were initially immersed into a mixture of acetone/water (7:3) for 30 ​min [40]. The treated scaffolds were incubated with chitosan solution and placed into the vacuum oven at room temperature for 30 ​min, following by rinsing three times with deionized water to remove the excess chitosan. After that, the scaffolds were suspended in gelatin solution for 30 ​min and washed with deionized water. The process was repeated for another cycle to obtain two bilayers of chitosan/gelatin coating onto the scaffolds [41]. Finally, the polyelectrolytes-modified scaffolds (PCG and SrHA@PCG) were freeze-dried and stored at −20 ​°C for further use.

### 2.4 Preparation of BMP-2 loaded scaffolds

BMP-2 was loaded onto the PCG and SrHA@PCG scaffold by a dripping and lyophilizing method [[42], [43], [44]]. Briefly, 50 ​μL or 100 ​μL of BMP-2 solution, with a concentration of 20 ​μg/mL, was dropped onto the PCG and SrHA@PCG scaffold and then followed by maintenance at room temperature for 4 ​h to obtain the BMP-2 loaded scaffolds (BMP-2@PCG and BMP-2/SrHA@PCG).

### 2.5 Characterization of SrHA and scaffolds

The morphology and structure of SrHA were characterized by transmission electron microscopy (TEM, JEM-2100 ​F, Jeol Ltd., Japan) at 200 ​kV. The average length of nanoparticles was calculated using ImageJ 1.34 software (National Institutes of Health, USA) from the TEM images. The scaffolds were sputtered with gold at 10 ​mA current for 30 ​s and the morphology was observed by scanning electron microscope (SEM, Phenom ProX, Phenom-World, Netherlands) at 10 ​kV. The average pore diameter was obtained from SEM images with ImageJ 1.34 software, where at least 50 measurements were randomly selected. The elements distribution was qualitatively analyzed with a Quantax 400 energy dispersive X-ray spectroscope (EDS, Bruker, Germany). Attenuated total reflectance–Fourier transform infrared (ATR–FTIR) spectra were performed with a PerkinElmer Spectrum Two spectrometer (Waltham, MA, USA) at a resolution of 4 ​cm^−1^ in the range of 400–4000 ​cm^−1^. X-ray diffraction (XRD) patterns were recorded on a D/max-2500 ​P ​C diffractometer (Rigaku Co., Japan) using Cu/K*a* radiation at 2θ range of 5–70°. Three specimens were tested for each sample.

### 2.6 Hydrophilicity characterization

#### 2.6.1 Water retention

The cylindrical scaffolds were prepared including PPP, SrHA@PPP and SrHA@PCG. Before immersion in deionized water, all the scaffolds were weighted and recorded as W_dry_. In order to assess the contribution of polyelectrolytes modification on the improvement of water absorption ratio of SrHA@PCG scaffold, pre-wetted PPP and pre-wetted SrHA@PPP scaffolds were prepared. PPP and SrHA@PPP scaffolds were treated with 75% ethanol for wetting. After rinsing with deionized water for several times, filter papers were used to acutely adsorb the residual water on the wetted PPP and SrHA@PPP scaffolds. Afterward, the dry PPP, pre-wetted PPP, pre-wetted SrHA@PPP and dry SrHA@PCG scaffolds were soaked in deionized water for 3 ​h, and then taken out to remove the excess water using filter papers. The weight of the samples was measured and recorded as W_wet_. Three specimens were measured for each sample. The water absorption ratio was calculated according to the following equation:

(1) Water absorption ratio ​= ​[(W_wet_ - W_dry_) / W_dry_] ​× ​100%

#### 2.6.2 Wettability and water permeability performance

The wettability of PPP, SrHA@PPP and SrHA@PCG scaffolds was detected by measuring the static water contact angles at room temperature. For water permeability evaluation, the front sides of SrHA@PCG scaffolds with different thickness (thickness: approximately 2.0 ​mm and 1.5 ​mm) were treated with 20 ​μL of FITC-BSA. After 30 ​min, the reverse sides of the samples were photographed.

### 2.7 Mechanical and degradation properties

Cylindrical samples with approximately 8 ​mm diameter and 6 ​mm height were prepared. The mechanical properties of PPP and SrHA@PCG scaffolds were measured using a mechanical tester (HY–940FS, Shanghai Heng Yu Instrument Co., Ltd., China) with 200 ​N load cell. The loading rate was set at 1 ​mm/min for all cylindrical samples. Three replicates were carried out for each group.

The degradation test was carried out by immersing the SrHA@PCG scaffold into PBS (pH ​= ​7.4) for 4, 6, 8, 12 and 16 weeks. After incubation for predetermined time points, the samples were taken out and washed twice with deionized water. The weight of the samples was weighed after freeze-drying and the weight loss was calculated at different time intervals according to the following equation:

(2) Weight loss ratio ​= ​[(W_0_ - W_t_) / W_0_] ​× ​100%

Where W_0_ is the initial weight of samples and W_t_ is the weight at different time points after freeze-drying. Three specimens were measured for each sample.

### 2.8 Protein immobilization capacity

First, different concentrations of FITC-BSA were prepared, including 100, 200 and 300 ​μg/mL. To study the contribution of polyelectrolytes modification on the improvement of protein immobilization capacity of SrHA@PCG scaffold, pre-wetted PPP and pre-wetted SrHA@PPP scaffolds were prepared as mentioned above. Then the dry PPP, pre-wetted PPP, pre-wetted SrHA@PPP and dry SrHA@PCG scaffolds were soaked in the 200 ​μg/mL FITC-BSA for 12 ​h. After that, the protein-loaded samples were taken out and washed twice with PBS, and the solution was entirely collected. The amount of free FITC-BSA in the collected solution was measured at the wavelength of 495 ​nm through a microplate reader (Multiskan GO, Thermo Scientific). Following a calibration curve, the amount of loaded protein on the scaffolds was determined and the loading weight relative to the volume of scaffolds was calculated according to the following equation:

(3) Loading weight ​= ​[(weight of used protein - weight of unloaded protein) / volume of scaffold] ​× ​100%

For the evaluation of protein immobilization capacity, SrHA@PCG scaffold was immersed into different concentrations of FITC-BSA (100, 200 and 300 ​μg/mL). After incubation for 12 ​h, the supernatant was removed and the samples were washed with PBS. Three replicates were performed for each group. Finally, the loading weight relative to the volume of scaffold and the loading weight percentage were calculated according to the unloaded amount of FITC-BSA using the following equation:

(4) Loading weight percentage ​= ​[(amount of used protein - amount of unloaded protein)/ amount of used protein] ​× ​100%

### 2.9 In vitro drug release

For BMP-2 protein release determination, BMP-2 loaded scaffold was placed into a tube containing 2 ​mL of PBS (pH ​= ​7.4). Then the tubes were maintained in an incubator and shaken at 37 ​°C with the speed of 100 ​rpm/min. At the predetermined time points, the supernatant was extracted and supplemented with fresh PBS. The BMP-2 concentrations in the collected solutions were detected using a BMP-2 ELISA kit (NeoBioscience, Shenzhen, China). The cumulative release percentage of BMP-2 from the scaffold at different time points was calculated. Three specimens were tested for each sample.

To investigate the release behavior of Sr ions, SrHA-doped scaffold was kept in a tube containing 3 ​mL of PBS, and then placed in a shaker under the conditions of 37 ​°C and 100 ​rpm/min 2 ​mL of PBS was extracted at the predetermined time points and then supplemented with 2 ​mL of fresh PBS. The concentration of Sr ions of extracted solution was monitored by inductively coupled plasma-atomic emission spectrometer (ICP-AES, Leeman, USA). Three replicates were performed in each time point.

### 2.10 In vitro biocompatibility

#### 2.10.1 CCK-8 assay

The cell proliferation on different scaffolds was evaluated by the CCK-8 assay. Bone marrow mesenchymal stem cells (BMSCs) were isolated from the femur of Sprague Dawley (SD) rat and cultured with DMEM/F-12 medium. Briefly, 5 ​× ​10^4^ ​cells/mL of BMSCs suspension was seeded on PPP, PCG and SrHA@PCG scaffolds in a 24-well cell culture plate, and then cultured in an incubator at 37 ​°C and 5% CO_2_. The CCK-8 working solution was prepared by diluting 10 times of stock solution with cell medium without FBS and penicillin-streptomycin. After incubation for 1, 3 and 7 days, the medium was removed and replaced with the CCK-8 working solution in each well. 100 ​μL of the supernatant was extracted and added into a 96-well plate, and the absorbance value of each well was measured by microplate reader at 450 ​nm.

#### 2.10.2 Fluorescent and SEM observation

After 3 and 7 days of culture, the medium in the cell culture plate was discarded and the cells were washed three times with PBS. Then, calcein-AM working solution was prepared and added into each well. After incubation for 30 ​min, the cells on different scaffolds were observed by inverted fluorescence microscope (Olympus IX71, Japan). When the cells were cultured on the scaffolds for 30 days, fluorescence images were also obtained by the calcein-AM staining. Besides, the cells were fixed with 4% paraformaldehyde for 30 ​min and then dehydrated with gradient ethanol solution. Finally, the samples were dried at the room temperature and the cells were imaged by SEM.

### 2.11 In vivo biocompatibility

The *in vivo* biocompatibility was assessed through a subcutaneous implantation model in ICR mice. The animal experiments were performed in accordance with the Guidelines for Care and Use of Laboratory Animals and approved by the Animal Ethics Committee of Shanghai Ninth People's Hospital, Shanghai Jiao Tong University School of Medicine. The 6-week-old male ICR mice were anesthetized by an intraperitoneal injection of 1% pentobarbital sodium. Then the sterile scaffolds were implanted into the subcutaneous pocket under the dorsum of mice. After post-operation for 4 and 8 weeks, the specimen were collected and fixed in 4% paraformaldehyde. The sections of 5-μm thickness were created and stained with hematoxylin and eosin (H&E) for tissue formation observation and Masson's trichrome for collagen formation observation. Additionally, the microvessels formation within the scaffolds was characterized by immunofluorescence staining of platelet endothelial cell adhesion molecule-1 (CD31) and the cell nucleus was stained with 4′,6-Diamidino-2-phenylindole dihydrochloride (DAPI). The quantitative analysis of fluorescence area of CD31 was accomplished using ImageJ software.

### 2.12 Osteogenic differentiation evaluation

The osteogenic effect of different scaffolds was firstly investigated by alkaline phosphatase (ALP) activity determination. Briefly, the cells were seeded on PCG, SrHA@PCG, BMP-2@PCG and BMP-2/SrHA@PCG scaffolds at a density of 1 ​× ​10^5^ per well. After 24 ​h of culture, the medium was replaced with osteogenic medium (DMEM/F12 medium supplemented with 50 ​μg/mL l-ascorbic acid, 10 ​mM β-glycerol phosphate and 10^−8^ ​M dexamethasone). At 7 and 14 days, the cells were rinsed with PBS and lysed with cell lysis buffer to leak the alkaline phosphatase. By centrifugation, the supernatant was taken out for ALP level detection by following the manufacturer's protocol. The content of total protein of each sample was also measured according to the instruction of BCA Protein Assay Kit. The ALP activity was normalized to total protein and expressed as μmol/min/mg protein.

The expression of osteo-related genes was detected to evaluate the osteogenic potential of different scaffolds. BMSCs were cultured on each scaffold and incubated for 7 days. Afterward, osteo-related genes were measured by quantitative real-time PCR (qRT-PCR) analysis, including runt-related transcription factor 2 (RUNX2), ALP, osteopontin (OPN) and osteocalcin (OCN). The total RNA of BMSCs in each sample was enriched using TRIzol reagent (Invitrogen, USA). NanoDrop 2000 spectrophotometer (Thermo Scientific, USA) was used to determine the concentration of total RNA. The single-stranded cDNA was synthesized using a Hieff™ First Strand cDNA Synthesis Kit (Yeasen, China) according to manufacturer's instructions. qRT-PCR was carried out by employing Hieff™ qPCR SYBR Green Master Mix Kit (Yeasen, China) on the Fast Real-Time PCR System (Applied Biosystems 7500, USA). The information of gene-specific primers was provided in Table S1. The qRT-PCR amplifications were performed under 95 ​°C for 5 ​min, 40 cycles of 95 ​°C for 10 ​s, and 60 ​°C for 30 ​s. The relative expression of target genes was calculated using the 2^−ΔΔCt^ method.

### 2.13 Rat calvarial bone defect model

8-week-old male SD rats were received from Shanghai SLAC Laboratory Animal Co. Ltd. (Shanghai, China). The calvarial defect model was created to evaluate the bone regeneration efficacy of PCG, SrHA@PCG, BMP-2@PCG and BMP-2/SrHA@PCG scaffolds. The rats were divided into five groups (n ​= ​6), including control group (without scaffold implantation) and scaffold implantation groups. After anesthetization with 3% pentobarbital sodium, 5-mm diameter defects were made on the rats’ skull and then inserted with the samples.

### 2.14 Micro-CT scanning and histological staining

After implantation for 8 and 12 weeks, the rats were sacrificed, and the specimens were harvested and fixed in 4% paraformaldehyde for micro-CT analysis and histological evaluation. The *in vivo* bone regeneration in the defect sites was analyzed on a micro-CT scanner (SkyScan 1176, Bruker). The 3D images of reconstructed bone tissues in the defect area were carried out for observation using CTvox software. The regions of interest were selected (height ​= ​1.5 ​mm and radius ​= ​2.5 ​mm) around the edge of defect area. The bone mineral density (BMD) and bone volume/tissue volume (BV/TV) in the regions of interest were calculated using the CTAn software to quantitatively analyze the newly formed bone within the defects. For the histological evaluation, the specimens were decalcified in 10% ethylenediaminetetraacetic acid (EDTA) and embedded in paraffin. The specimens were sectioned (5-μm thickness) and stained with H&E and Masson's trichrome. Immunofluorescence staining of CD31, α-smooth muscle actin (α-SMA) and OCN was performed to evaluate the vascularization and bone formation. The quantitative data of positively-stained areas were carried out using ImageJ software.

### 2.15 Statistical analysis

The experimental data are reported as mean ​± ​standard deviation. The one-way analysis of variance (ANOVA) with the post hoc Tukey's method was performed to evaluate the statistically significant differences using OriginPro 2016 (OriginLap Cor., USA). The values of ​∗*P* ​< ​0.05 and ∗∗*P* ​< ​0.01 were considered to indicate statistical significance.

## 3 Results and discussion

### 3.1 Characterization of scaffolds

**Fig. 2:**
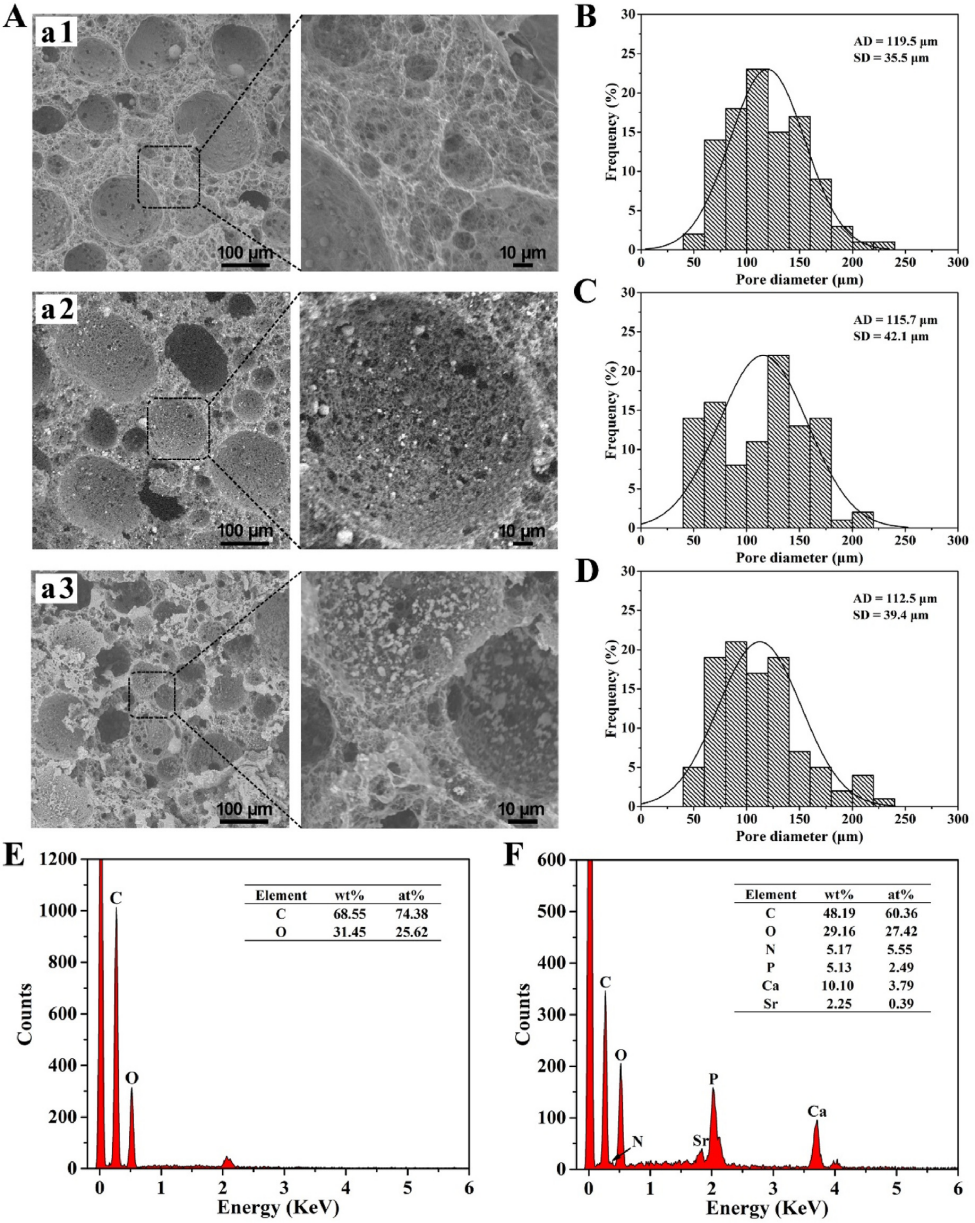
(A) SEM images of PPP, SrHA@PPP and SrHA@PCG scaffolds, a1: PPP scaffold, a2: SrHA@PPP scaffold, a3: SrHA@PCG scaffold, and the enlarged photographs were on the right. (B–D) The pore size distributions of (B) PPP, (C) SrHA@PPP and (D) SrHA@PCG scaffolds. (E, F) EDS patterns of (D) PPP and (E) SrHA@PCG scaffolds.

Before the composite scaffold fabrication, nanometer-sized SrHA was prepared by hydrothermal method and observed by TEM. SrHA showed rod-like shape with 141.0 ​± ​32.2 ​nm in length (Fig. S1), which was similar to the nanohydroxyapatite [6,8]. The microstructure of as-prepared scaffolds was characterized by SEM. As shown in Fig. 2A, both PPP scaffold and SrHA@PCG scaffold exhibited obvious spherical macroporous structure. Through the enlarged photographs, nanofibrous structure can be clearly observed for PPP scaffold, SrHA@PPP scaffold and SrHA@PCG scaffold. The pore size distribution within the scaffolds was calculated from the SEM images using ImageJ software. It was measured to be 119.5 ​± ​35.5 ​μm for the bare PPP scaffold (Fig. 2B). As for the SrHA@PPP scaffold and SrHA@PCG scaffold, the pore size was calculated to be 115.7 ​± ​42.1 ​μm and 112.5 ​± ​39.4 ​μm (Fig. 2C and D), respectively. By contrast, there was no significant difference on the pore size between PPP scaffold, SrHA@PPP scaffold and SrHA@PCG scaffold, which meant that the addition of SrHA did not have apparent influence on the microstructure of scaffold. We found that the SrHA particles was located within the scaffolds. Interestingly, many smaller pores were distributed in the large pores of scaffolds, reflecting the hierarchical porous structure. It has been reported that the highly porous structure within the scaffolds can facilitate nutrients transport (permeability and diffusion), gas exchange and ingrowth of tissues compared with the dense scaffolds [23,45]. Therefore, a highly porous scaffold was fabricated by the TIPS method, which can allow the mass exchange throughout the scaffold.

Fig. 2E and F shows the EDS patterns of PPP scaffold and SrHA@PCG scaffold, which was analyzed according to different characteristic energy of X-ray signals of different elements. As can be seen from Fig. 2E, PPP scaffold was mainly featured with the C and O elements in terms of elements composition. Notably, in addition to the C and O elements, SrHA@PCG scaffold displayed the characteristic signals of N, P, Ca and Sr elements (Fig. 2F). The appearance of P, Ca and Sr elements was ascribed to the incorporation of SrHA in the scaffold. In addition, the evidence of N element was detected in the SrHA@PCG scaffold, which was attributed to the successful coating of polyelectrolytes. The results indicated that SrHA could be successfully incorporated into PPP scaffold and the scaffold could be successfully modified with chitosan/gelatin layers.

**Fig. 3:**
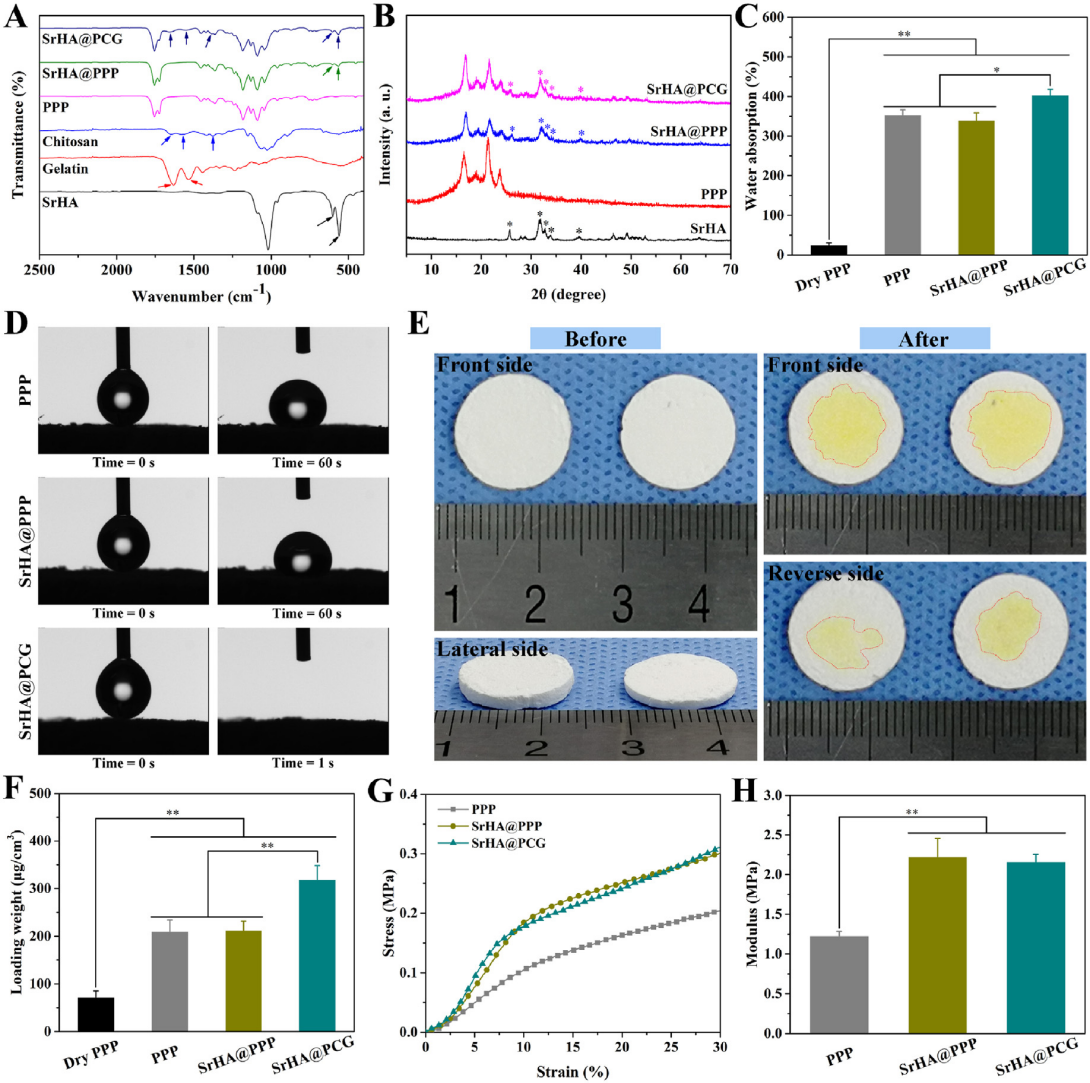
(A) FTIR spectra of different samples. (B) XRD patterns of different samples, ∗ indicated the characteristic peaks of HA. (C) The water absorption ratios of dry PPP, pre-wetted PPP, pre-wetted SrHA@PPP and dry SrHA@PCG scaffolds. (D) The photographs captured from the contact angle test after water droplets contacting with PPP, SrHA@PPP and SrHA@PCG scaffolds. (E) The permeability performance evaluation of SrHA@PCG scaffolds by adding with FITC-BSA in the front side, the thickness of scaffolds was approximately 2.0 ​mm (left) and 1.5 ​mm (right), respectively. (F) The protein loading capacities of dry PPP, pre-wetted PPP, pre-wetted SrHA@PPP and dry SrHA@PCG scaffolds. (G) Stress-strain curves of different scaffolds under dry state. (H) Compression modulus of different scaffolds under dry state. ∗*P* ​< ​0.05, ∗∗*P* ​< ​0.01.

Next, the polyelectrolytes coating and SrHA incorporation were verified by FTIR spectra and XRD patterns. Fig. 3A shows the FTIR spectra of different samples, including SrHA, gelatin, chitosan, PPP scaffold, SrHA@PPP scaffold and SrHA@PCG scaffold. According to the spectrum of SrHA, the characteristic peaks at 560 and 600 ​cm^−1^ were attributed to the bending of the PO_4_^3−^ group [46]. In the gelatin sample, the peaks at 1538 and 1632 ​cm^−1^ were ascribed to the N–H bending vibration (amide Ⅱ) and C=O stretching vibration (amide Ⅰ) in the amide of gelatin [47]. From the spectrum of chitosan, the characteristic peaks of C–O stretching, N–H bending and C–H bending vibration were appeared at 1645, 1577 and 1376 ​cm^−1^ [48]. Compared with the PPP scaffold, the characteristic peak of PO_4_^3−^ group was appeared in the SrHA@PPP sample, indicating the blending of SrHA. Meanwhile, the absorption peak of PO_4_^3−^ group corresponding to SrHA was also observed in the SrHA@PCG sample. Moreover, new absorption peaks located at 1552 and 1658 ​cm^−1^, which were assigned to NH_2_ and CONH_2_ groups, could be clearly observed in the SrHA@PCG sample. Compared with the spectra of gelatin and chitosan, these absorption peaks in SrHA@PCG were slightly shifted, which may be caused by the interaction between gelatin and chitosan. Fig. 3B shows the XRD patterns of SrHA, PPP, SrHA@PPP and SrHA@PCG scaffolds. From the XRD pattern of SrHA, the diffraction planes of (002), (211), (300), (202) and (310) were emerged at 26.0°, 31.9°, 32.9°, 33.9° and 39.8°, which were consistent with the characteristic diffraction peaks of hydroxyapatite [6]. The characteristic diffraction peaks of SrHA were not appeared in the sample of PPP scaffold, but could be observed in the SrHA@PPP and SrHA@PCG samples, indicating that SrHA was successfully incorporated into the composite scaffolds. The above results demonstrated the successful preparation of SrHA-incorporated scaffold and subsequent polyelectrolytes modification.

The hydrophilicity of scaffolds was closely associated with the transportation of nutrients, cell attachment and cell morphology on the surface [27,49,50]. Thus, the hydrophilic performance has been considered a very important characteristic to the biological properties of scaffold. Fig. 3C shows the water absorption ratios of different scaffolds. It was noted that the dry PPP scaffold presented the least amount of water absorption, with a water absorption ratio of only 25.47 ​± ​4.83%. By contrast, the pre-wetted PPP and SrHA@PPP scaffolds showed better water absorption performance compared with the dry PPP scaffold, with water absorption ratios of 353.03 ​± ​12.92% and 339.56 ​± ​19.14%, respectively. In terms of water absorption ratio, there was no significant difference between them. However, the water absorption ratio of dry SrHA@PCG scaffold (403.76 ​± ​14.70%) was the highest, which was 15.8-fold higher than that of dry PPP scaffold, and which was also significantly higher than that of pre-wetted PPP and SrHA@PPP scaffolds (*P* ​< ​0.05). The result indicated that the surface modification of chitosan/gelatin significantly enhanced the water absorption property of scaffolds. Subsequently, the hydrophilicity was further characterized by contact angle test. As shown in Fig. 3D, the water drop can keep the shape integrally on the surface of PPP and SrHA@PPP scaffolds. The contact angles were measured to be more than 125° after contacting for 60 ​s. However, the contact angle was decreased to 0° for SrHA@PCG scaffold when detected at a very short time (<1 ​s), revealing a fully wetted state. The water drop can completely penetrate the inside of the scaffold, suggested the excellent hydrophilicity of SrHA@PCG scaffold. Furthermore, the hydrophilicity of SrHA@PCG scaffold was confirmed by the permeability performance evaluation (Fig. 3E). It was observed that FITC-BSA can pass through from one side to the opposite side of SrHA@PCG scaffold, revealed that the porous structure was interconnected and allowed nutrients permeation. These results indicated that the hydrophilicity of SrHA@PPP scaffold was greatly improved after modification of polyelectrolytes, and SrHA@PCG scaffold had excellent hydrophilicity.

To investigate the protein adsorption performance of scaffolds, BSA protein was employed to evaluate the binding capacity. The better protein immobilization efficiency can carry much more bioactive factors so that the biological function of scaffolds can be improved. As shown in Fig. 3F, the protein loading weight of dry PPP scaffolds was only 72.09 ​± ​13.54 ​μg/cm^3^. After wetting treatment, the loading weights of PPP scaffold and SrHA@PPP scaffold were increased to 210.01 ​± ​24.18 ​μg/cm^3^ and 212.21 ​± ​19.39 ​μg/cm^3^, respectively. With the chitosan/gelatin modification, the dry SrHA@PCG scaffold showed the best protein loading performance (318.63 ​± ​29.37 ​μg/cm^3^), which was 4.4-fold, 1.5-fold and 1.5-fold higher than that of dry PPP scaffold, treated PPP and SrHA@PPP scaffolds, respectively. Furthermore, the protein binding efficiency of SrHA@PCG scaffold was studied by soaking in different concentrations of BSA (Fig. S2). We found that the loading weight of BSA on the SrHA@PCG scaffold was gradually increased with the increasing protein concentration, while the loading weight percentage of BSA was decreased from 51.72 ​± ​3.20% (100 ​μg/mL) to 36.04 ​± ​3.32% (200 ​μg/mL) and 32.92 ​± ​2.28% (300 ​μg/mL). These results indicated that the chitosan/gelatin modification can not only improve the hydrophilicity of scaffolds, but also facilitate the permeation of protein solution and enhance the protein immobilization efficiency.

The mechanical properties of the prepared PPP, SrHA@PPP and SrHA@PCG scaffolds were tested. As seen from the stress-strain curves in Fig. 3G, the incorporation of SrHA can significantly enhance the compressive strength, showing the compressive stress increased from 0.13 ​± ​0.05 ​MPa to 0.16 ​± ​0.02 ​MPa. Accordingly, the compression modulus of SrHA@PCG scaffold (2.16 ​± ​0.09 ​MPa) was higher than that of PPP scaffold (1.21 ​± ​0.03 ​MPa), showing an increase of about 1.8-fold (Fig. 3H). Additionally, the compressive performance of different scaffolds under a wetted state was also investigated, as shown in Fig. S3. It also demonstrated that the compressive strength of SrHA@PPP and SrHA@PCG scaffolds was higher than that of PPP scaffold (*P* ​< ​0.01). Therefore, the incorporation of SrHA can significantly improve the mechanical properties of scaffolds. The degradation property of SrHA@PCG scaffold were investigated by immersion in PBS for 16 weeks (Fig. S4). From the degradation curve, it was observed that SrHA@PCG scaffold showed a gradual decrease in weight over time. After incubation for 16 weeks, the weight loss ratio of SrHA@PCG scaffold was 23.45 ​± ​1.39%. Therefore, the result of degradation test demonstrated that the SrHA@PCG scaffold was degradable.

The release behavior of BMP-2 protein from BMP-2@PCG was examined by soaking in PBS at different time points. As seen from Fig. S5A, it showed that the fast release was appeared at the initial time, with 21.2% of released BMP-2 at 24 ​h, and then followed by a sustained release profile in the following release time. The initially rapid release was strongly attributed to the leakage of BMP-2 from scaffold surface. Because the permeation of BMP-2 throughout the scaffold, it allowed the delayed release and thus resulting in a prolonged release formulation subsequently. As shown in Fig. S5B, the leakage of Sr ions from SrHA@PCG scaffold was also tested, which released in a continuous release manner. With the increase of soaking time, the released Sr ions was gradually increased. Thus, the PCG scaffold allowed an early rapid release of adsorbed protein, while maintained a long-term sustained release of Sr ions.

### 3.2 Biocompatibility of scaffolds

**Fig. 4:**
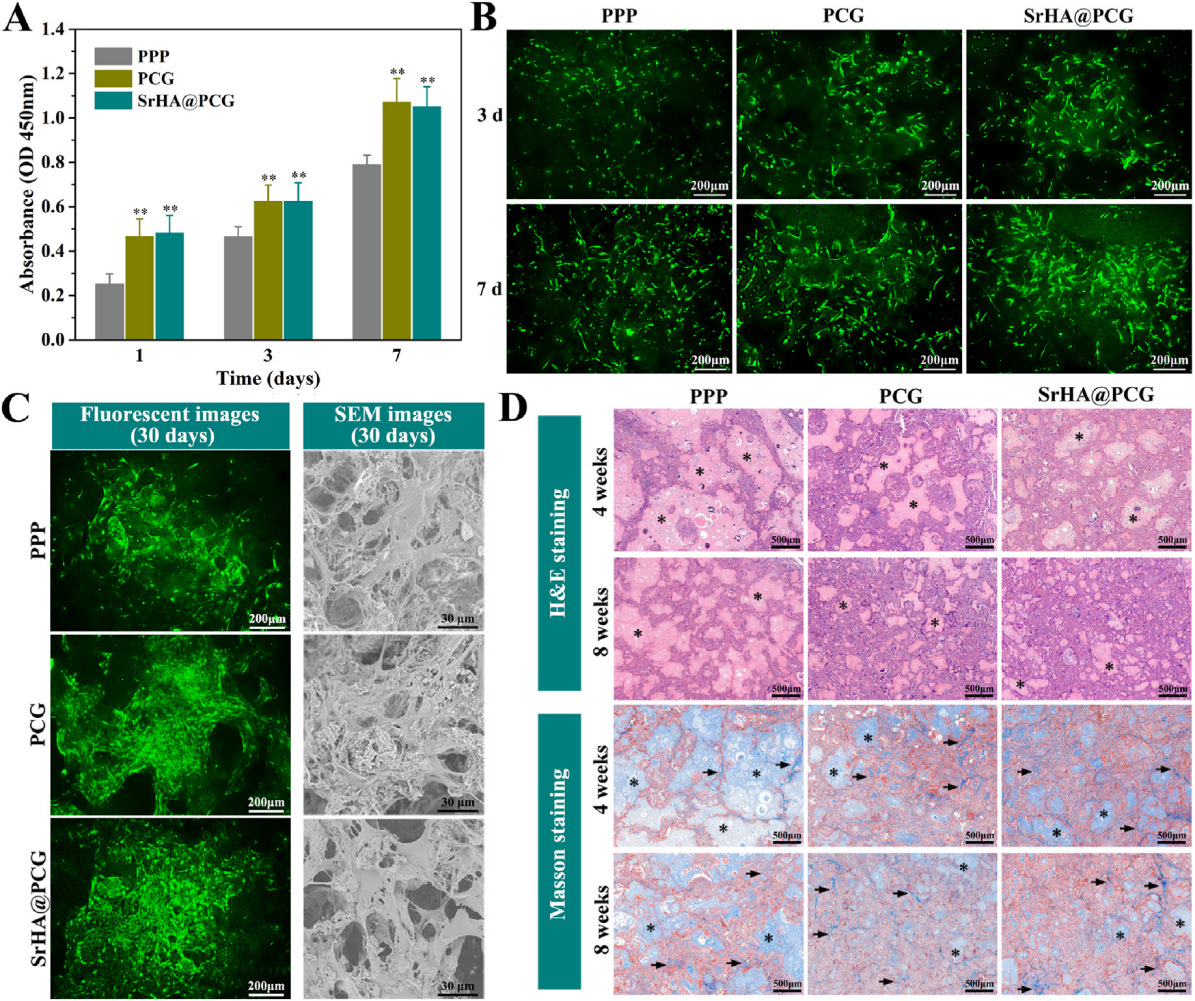
(A) Cells proliferation on PPP, PCG and SrHA@PCG scaffolds after BMSCs seeding for 1, 3 and 7 days. (B) Fluorescent images of BMSCs cultured on PPP, PCG and SrHA@PCG scaffolds for 3 and 7 days after Calcein-AM staining. (C) Fluorescent images and SEM images of BMSCs cultured on PPP, PCG and SrHA@PCG scaffolds for 30 days, the fluorescent images were obtained from calcein-AM staining. (D) H&E and Masson’ staining of PPP, PCG and SrHA@PCG scaffolds after 4 and 8 weeks of subcutaneous implantation, the asterisks indicated the materials and black arrows indicated the collagen fiber. ∗∗*P* ​< ​0.01, compared to PPP group.

The *in vitro* biocompatibility of composite scaffolds was firstly investigated by CCK-8 assay. As shown in Fig. 4A, the cells grown on PPP scaffold, PCG scaffold and SrHA@PCG scaffold showed obvious proliferation as the culture time increased from 1 to 7 days. However, the cell number on the PCG scaffold and SrHA@PCG scaffold was significantly higher than that of PPP scaffold at each culture time point, while there were no significant differences between the groups of PCG scaffold and SrHA@PCG scaffold. The cells cultured on the PPP, PCG and SrHA@PCG scaffolds were also observed by live cells staining. Fig. 4B shows the fluorescent images of BMSCs grown on different scaffolds at 3 and 7 days. It can be seen that more cells with spread morphology were adhered on the surface of scaffolds over time. Notably, larger number of cells were in spread morphology when cultured on PCG scaffold and SrHA@PCG scaffold at 7 days as compared with cultured on PPP scaffold. Furthermore, a longer time of cells culture on different scaffolds was observed by fluorescent and SEM images. As shown in Fig. 4C, the surface of scaffolds was covered with a large number of cells after 30 days of culture, whereas more cells were observed on the PCG scaffold and SrHA@PCG scaffold. These results indicated that the chitosan/gelatin-modified scaffold could significantly promote cell growth which was probably attributed to the favorable biosurface, such as excellent hydrophilicity, high affinity of protein.

The *in vivo* biocompatibility of composite scaffolds was studied by subcutaneous embedding through the H&E and Masson’ staining. As shown in Fig. 4D, after 4 weeks of implantation, the H&E staining showed that the adjacent tissue was penetrated into the PPP scaffold, PCG scaffold and SrHA@PCG scaffold, but the infiltrated tissues in the PPP scaffold were less distributed and a large area of residual scaffold materials could be observed. In contrast, the infiltration of subcutaneous tissues in PCG scaffold and SrHA@PCG scaffold was more evident than in PPP scaffold. At 8 weeks of implantation, much more tissues were observed for each scaffold group, while the more tissue infiltration was appeared in the groups of polyelectrolytes-modified scaffolds. As for the Masson’ staining, the same tendency of tissue ingrowth within the scaffolds was displayed. At the same time, it observed that more collagen fibers were formed within the PCG scaffold and SrHA@PCG scaffold. Therefore, the *in vivo* experiment suggested that the polyelectrolytes modification on the scaffolds exhibited better tissue infiltration and collagen fiber formation, which was beneficial to tissue regeneration.

**Fig. 5:**
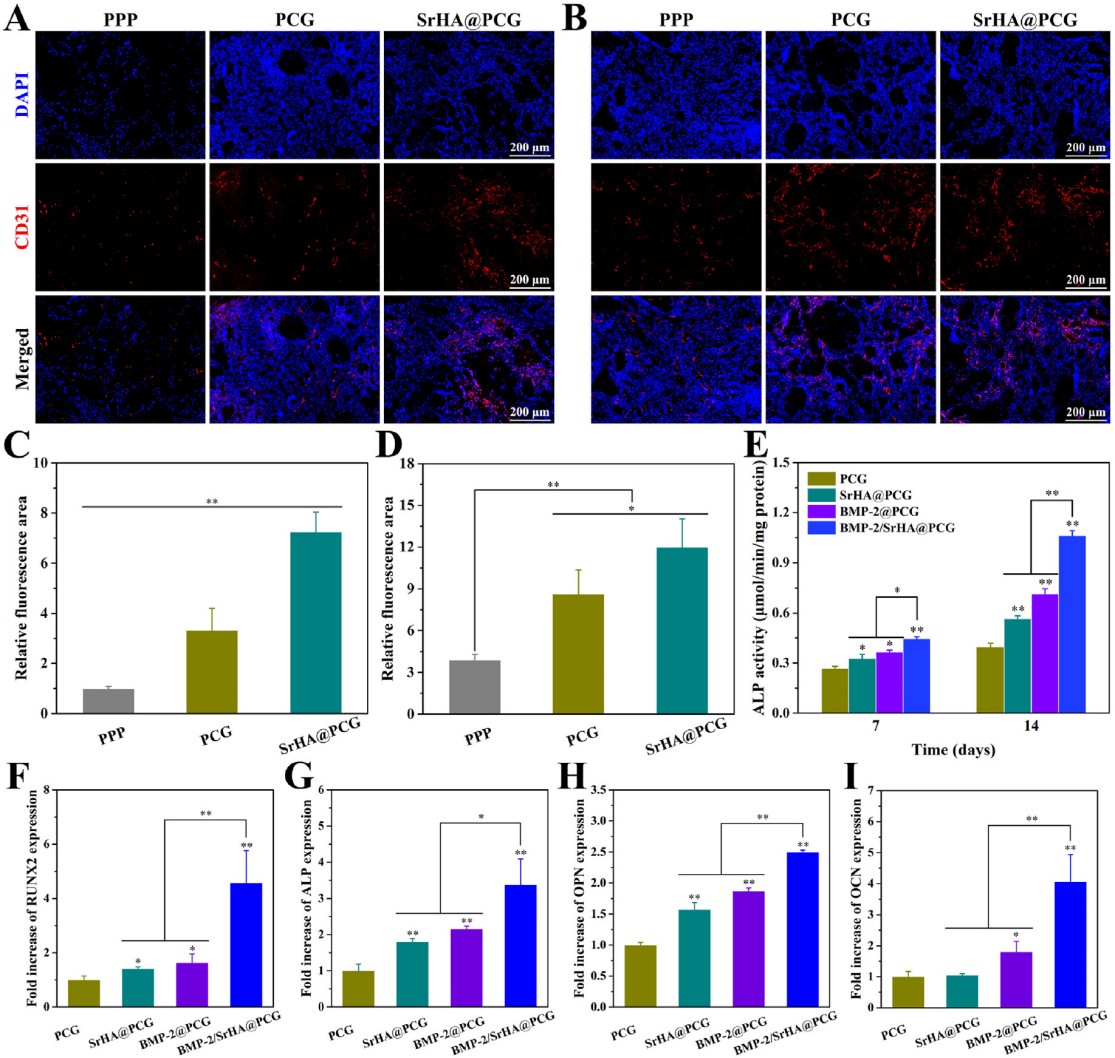
(A, B) Representative immunofluorescence images of CD31 (red) stained at (A) 4 weeks and (B) 8 weeks after subcutaneous implantation of PPP, PCG and SrHA@PCG scaffolds, and the cell nucleus was stained with DAPI (blue). (C, D) Quantitative analysis of positively-stained areas of CD31 at (C) 4 weeks and (D) 8 weeks. (E) ALP activity of BMSCs cultured on PCG, SrHA@PCG, BMP-2@PCG and BMP-2/SrHA@PCG scaffolds for 7 and 14 days. (F–I) Osteogenesis-related genes expression of BMSCs after incubation with PCG, SrHA@PCG, BMP-2@PCG and BMP-2/SrHA@PCG scaffolds for 7 days, including (F) RUNX2, (G) ALP, (H) OPN and (I) OCN. ∗*P* ​< ​0.05, ∗∗*P* ​< ​0.01, compared to PCG group. (For interpretation of the references to colour in this figure legend, the reader is referred to the Web version of this article.)

Thereafter, the vascularization within the implanted scaffolds was assessed by immunofluorescence staining of CD31. As shown in Fig. 5A, the specimens were appeared to be positively stained at 4 weeks, indicating the formation of vascular network within the scaffolds. However, the positively-stained areas of CD31 in the groups of PCG and SrHA@PCG were higher than that of PPP group (Fig. 5C). It was worth noticing that the SrHA@PCG group had more immunofluorescence and positively-stained areas, suggesting that the level of CD31 expression in SrHA@PCG group was significantly increased. After 8 weeks, the CD31 expression among the groups was more evident as compared with 4 weeks (Fig. 5B). As expected, the SrHA@PCG group presented the highest positively-stained areas, indicating the best microvascular formation (Fig. 5D). In this regard, the chitosan/gelatin modification brought more tissue ingrowth throughout the scaffolds, which was corelated with the large amount of internally capillary network. Importantly, it was found that SrHA@PCG scaffold was able to stimulate more microvascular formation. The previous study had demonstrated that Sr-contained scaffolds could exhibit better angiogenic behavior (such as facilitating tube formation and angiogenesis-related gene expression of HUVECs) and enhance the *in vivo* vascularization by upregulating the levels of angiogenic factors expression [51]. Furthermore, other studies suggested that Sr-loaded scaffolds significantly promoted the blood vessels formation by modulating the transformation of macrophage phenotype from M1 to M2 and secretion of pro-angiogenic factors (such as PDGF-BB) [52,53].

### 3.3 Osteogenic effect of scaffolds

ALP is widely considered as an early marker of osteogenic differentiation of BMSCs, which is up-regulated in the early stage of osteogenesis [54]. Thus, the ALP activity of BMSCs was measured to characterize the osteogenic effect of scaffolds. Fig. 5E shows the results of ALP activity of BMSCs cultured on different scaffolds for 7 and 14 days. It can be seen that the levels of ALP activity in BMSCs cultured on PCG, SrHA@PCG, BMP-2@PCG and BMP-2/SrHA@PCG scaffolds were significantly increased when the incubation time was extended. At the same time, the expression level of ALP in BMSCs grown on SrHA@PCG scaffold, BMP-2@PCG scaffold and BMP-2/SrHA@PCG scaffold was higher than on PCG scaffold at 7 and 14 days. Compared with the SrHA@PCG and BMP-2@PCG groups, the BMP-2/SrHA@PCG group showed higher level of ALP activity. The result revealed that the ALP activity of BMSCs could be up-regulated by the stimulation of BMP-2 or SrHA-loaded scaffolds.

Furthermore, the osteogenic potential of composite scaffolds was examined by qRT-PCR analysis. The osteo-related genes expression, including RUNX2, ALP, OPN and OCN, were detected when BMSCs were seeded on different scaffolds for 7 days. Among these osteogenic markers, RUNX2 is a member of the RUNX family of transcription factors. During the stimulation of osteogenic factors, RUNX2 expression is up-regulated and involved in the early process of osteogenic differentiation and bone formation [54]. Following the upregulation of RUNX2, the downstream indicators such as ALP, OPN and OCN will be in elevated expression level. As shown in Fig. 5F–I, on the 7th day of culture, the mRNA levels of RUNX2, ALP, OPN and OCN in BMP-2@PCG group and BMP-2/SrHA@PCG scaffold group were higher than those in PCG group. For SrHA@PCG group, the increased mRNA levels of RUNX2, ALP and OPN were detected as compared with PCG group. Among these scaffolds, the BMP-2/SrHA@PCG scaffold significantly enhance the mRNA expression of osteo-related genes. These results suggested that the scaffolds with loading of BMP-2 or Sr ions had obvious promoting effect on osteogenic differentiation of osteoblasts, while the scaffold containing both of them possessed stronger osteogenic ability.

### 3.4 Effect of scaffolds on bone regeneration

**Fig. 6:**
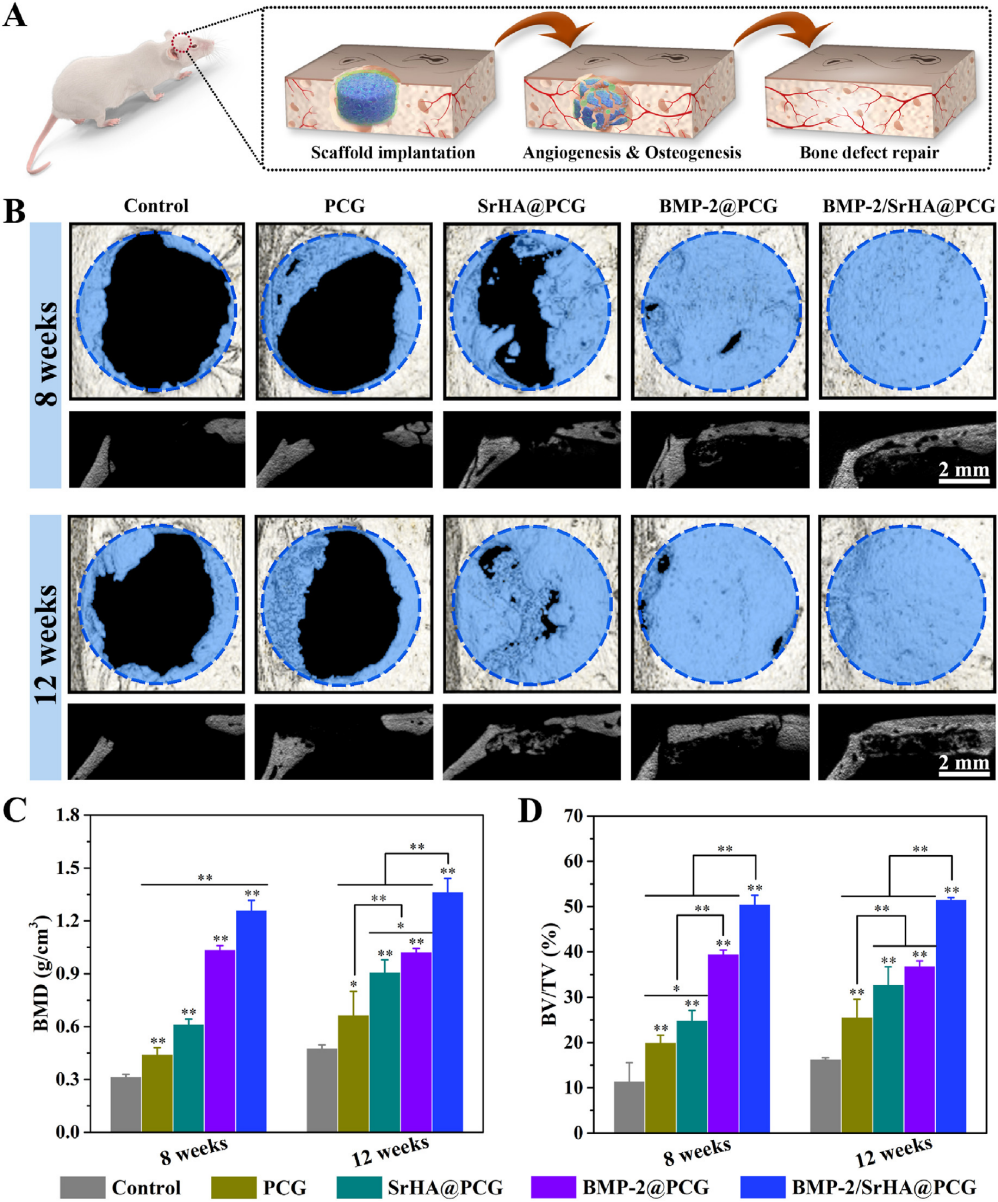
*In vivo* bone regeneration in a rat calvarial defect model. (A) Schematic illustration for rat calvarial defect repair by scaffolds implantation. (B) Representative 3D-reconstructed micro-CT images after implantation of PCG, SrHA@PCG, BMP-2@PCG and BMP-2/SrHA@PCG scaffolds for 8 and 12 weeks including transverse and coronal sections. (C, D) Quantitative analysis of microstructural parameters of regenerated bone tissues including (C) BMD and (D) BV/TV. The control group was conducted without scaffold implantation. ∗*P* ​< ​0.05, ∗∗*P* ​< ​0.01, compared to control group.

The *in vivo* bone regeneration after the implantation of different scaffolds was evaluated by applying a rat calvarial defect model (Fig. 6A). To precisely observe the newly formed bone tissue within the defect areas, the collected specimens were scanned with micro-CT system. The morphology and amount of new bone tissue within defects were analyzed by two-dimensional and three-dimensional images and micro-architectural parameters at 8 weeks and 12 weeks post-implantation. As shown in Fig. 6B, the areas of defect were obviously decreased for each group over the experimental period. In the control group, new bone tissue was regenerated from the edge towards the center of defect site. Although the size of the defect was found to be reduced from 8 weeks to 12 weeks, but still remaining a large region of defect for the control group, indicating the great challenge for self-healing when without treatment. While for the PCG group, it still showed little new bone formation surrounding the defects border. When the defect was treated with SrHA@PCG, large amount of new bone formation could be clearly observed. Compared with other groups, much more bone tissues were formed inside the defects after treated with BMP-2@PCG and BMP-2/SrHA@PCG, and the defects sites were totally covered with new bone tissues. However, there was no obvious difference of regenerated bone tissue between the two groups of BMP-2@PCG and BMP-2/SrHA@PCG when observed from the transverse views. From the coronal sections, it can be seen that more bone tissue grew into the BMP-2/SrHA@PCG scaffold, which was better than that of BMP-2@PCG group, indicating the preferable stimulatory effect on new bone formation of dual-factor delivery scaffold. Furthermore, the regenerated bone tissues were also quantitatively analyzed by BMD and BV/TV (Fig. 6C and D). At each time point, the values of BMD and BV/TV in the groups of SrHA@PCG, BMP-2@PCG and BMP-2/SrHA@PCG were significantly higher than those of the control and PCG groups. While the BMP-2/SrHA@PCG group exhibited the highest BMD and BV/TV at 12 weeks, which meant that the combination of BMP-2 and SrHA had the strongest bone regeneration ability.

**Fig. 7:**
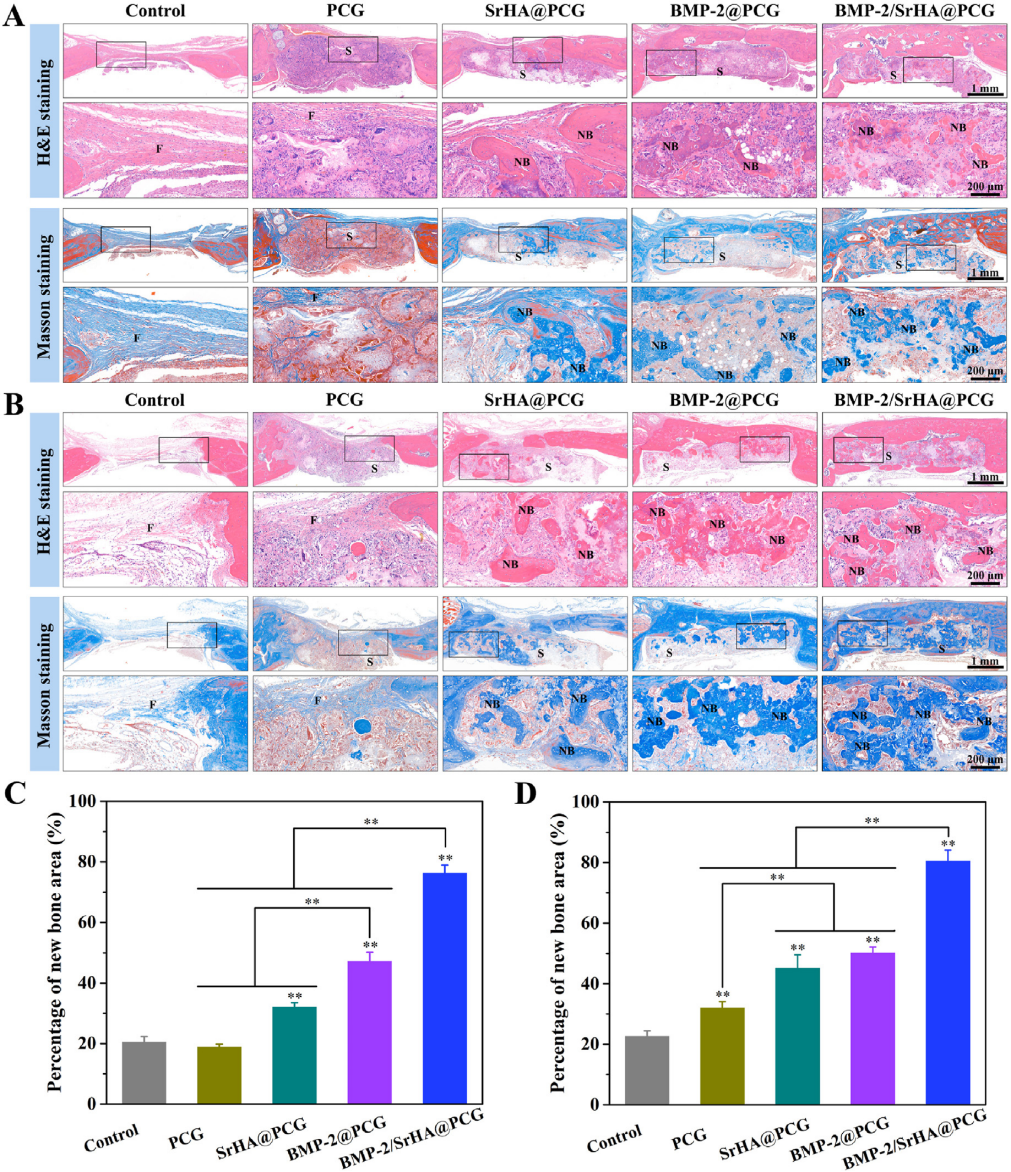
Histological evaluation of bone regeneration in a rat calvarial defect model. (A, B) Representative images of H&E staining and Masson's trichrome staining at (A) 8 weeks and (B) 12 weeks after implantation of PCG, SrHA@PCG, BMP-2@PCG and BMP-2/SrHA@PCG scaffolds (F, fibrous tissue; NB, newly formed bone tissue; S, scaffold residuals). (C, D) Percentages of newly formed bone area at (C) 8 weeks and (D) 12 weeks using ImageJ software for analysis. The control group was operated without scaffold treatment. ∗*P* ​< ​0.05, ∗∗*P* ​< ​0.01, compared to control group.

To further verify the osteogenic effect of these scaffolds on calvarial defect repair, histological staining was carried out at 8 weeks and 12 weeks (Fig. 7). According to the results of the H&E staining, the defect was occupied with a large amount of fibrotic tissue in the eighth week for the control group (Fig. 7A). At 12 weeks of implantation, control group displayed that only a few newly formed bone tissues were appeared at the edge of the defect area, while the middle of defect area was fully filled with fibrotic tissue (Fig. 7B). Similarly, there were no apparent bone tissues grew into the PCG scaffold. However, it was worth noting that the interior of PCG scaffold was fully infiltrated by fibrotic tissues. It suggested that PCG scaffold had great improvement of cell infiltration and extracellular matrix (ECM) deposition. Furthermore, it observed that other scaffold groups also presented abundant tissue infiltration, while the amount of residual scaffold materials were very low. Importantly, the new formed bone tissues were existed within the SrHA@PCG, BMP-2@PCG and BMP-2/SrHA@PCG scaffolds, which was strongly accompanied by the degradation of polymers and tissue ingrowth. As expected, the amount of new bone tissue was increased as the implantation time increased in all scaffold groups. The SrHA@PCG group only showed the limited new bone formation. Surprisingly, in the BMP-2@PCG and BMP-2/SrHA@PCG groups, a larger amount of new bone tissue was already distributed surrounding the scaffold implants due to the effective induction of released bioactive factors. In particular, the BMP-2/SrHA@PCG group exhibited the most bone tissue ingrowth from the adjacent area to the inside part of scaffold after 12 weeks postsurgery. As seen from the H&E images, the defect area in the BMP-2/SrHA@PCG group was well bridged by newly formed bone tissue. In addition, Masson's trichrome staining was also performed to confirm the bone repair capability of composite scaffolds. In accordance with H&E staining results, only the edge of the defect sites displayed a small fraction of new bone for the control and PCG groups. Notably, a large amount of regenerated bone tissue was observed in the groups of SrHA@PCG, BMP-2@PCG and BMP-2/SrHA@PCG scaffolds. While for the BMP-2@PCG and BMP-2/SrHA@PCG groups, the defect areas were almost filled with mineralized bone tissues. As expected, the BMP-2/SrHA@PCG group showed the most extensive mineralized bone and the thickest bone matrix, which not only generated at the surrounding area but also at the interior of the scaffold. Furthermore, the quantitative analysis of newly formed bone area at 8 weeks and 12 weeks revealed the same tendency (Fig. 7C and D). According to the regenerative microenvironment and biological function of as-prepared composite scaffold, intramembranous ossification occupied the predominant process for calvarial bone regeneration [55,56]. Overall, the histological results suggested that the BMP-2/SrHA@PCG scaffold had excellent pro-osteogenic effect to evoke a large amount of new bone tissue ingrowth.

**Fig. 8:**
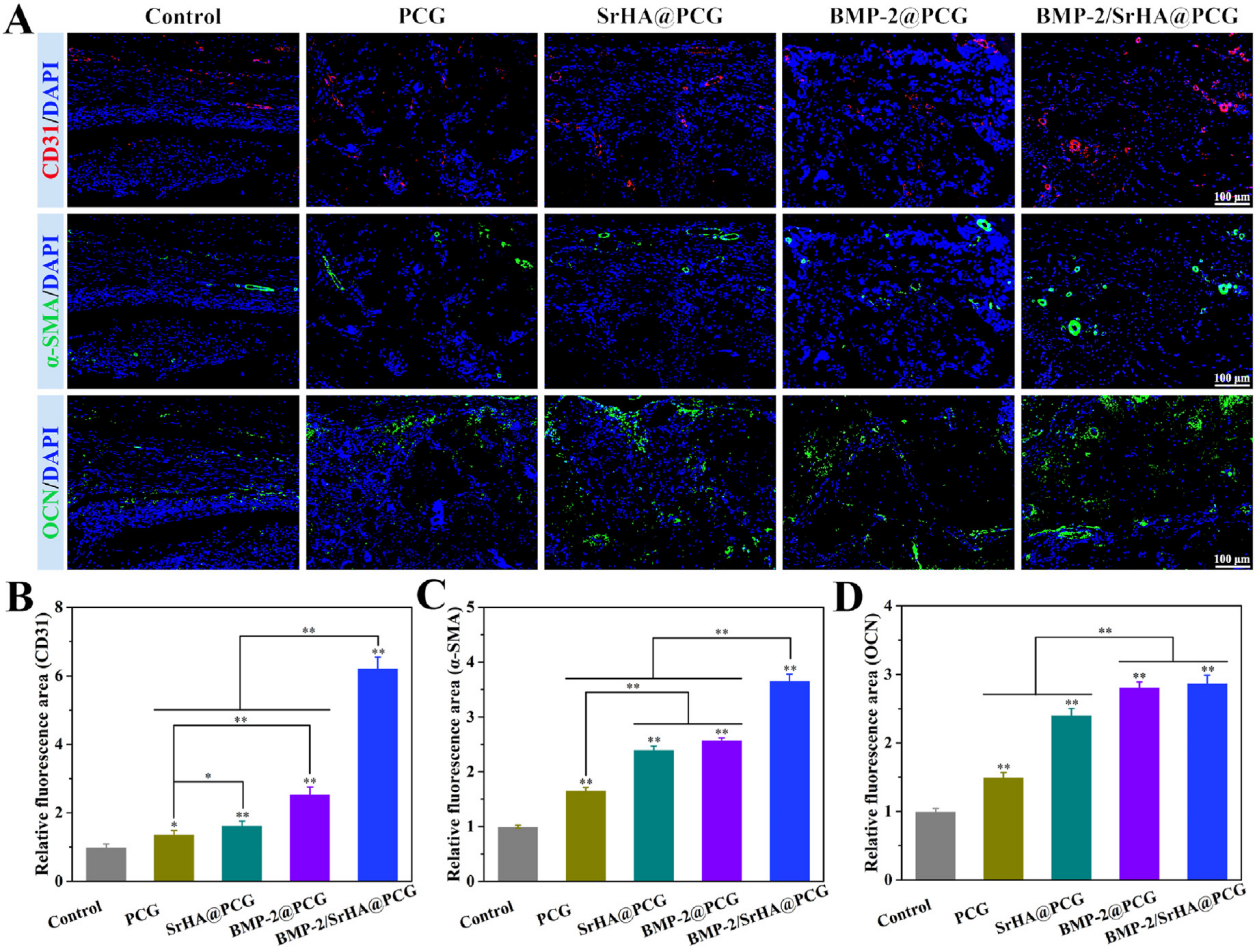
Histological evaluation of vascularization and bone regeneration by immunofluorescence staining. (A) Representative immunofluorescence images of CD31 (red), α-SMA (green) and OCN (green) stained at 8 weeks postoperatively after the bone defect sites implanted with PPP, PCG and SrHA@PCG scaffolds, and the cell nucleus was stained with DAPI (blue). (B–D) Quantitative analysis of positive areas of CD31, α-SMA and OCN expression. ∗*P* ​< ​0.05, ∗∗*P* ​< ​0.01, compared to control group. (For interpretation of the references to colour in this figure legend, the reader is referred to the Web version of this article.)

During the bone regeneration at the defect area, the formation of blood vessels was accompanied. In this regard, immunofluorescence staining of CD31 and α-SMA was carried out to evaluate the newly formed blood vessels after 8 weeks of scaffold implantation. The representative immunofluorescence images were depicted in Fig. 8A. It showed that SrHA@PCG, BMP-2@PCG and BMP-2/SrHA@PCG groups had remarkable positively-stained immunofluorescence of CD31 and α-SMA expression. While for the BMP-2/SrHA@PCG group, it yielded the more noticeable immunofluorescence of the two markers, indicating the higher degree of *in vivo* vascularization. The quantitative data of CD31 and α-SMA expression in all groups clearly supported the corresponding conclusions, where the BMP-2/SrHA@PCG group showed significantly higher fluorescence areas of CD31 and α-SMA staining (Fig. 8B and C). Furthermore, the expression of osteogenic marker was also detected by immunofluorescence staining, as provided in Fig. 8A and D. The result showed that the fluorescence areas of OCN expression in the BMP-2@PCG group and BMP-2/SrHA@PCG group were at a comparable level, but which were higher than that of other groups. Collectively, these results demonstrated that the BMP-2/SrHA@PCG scaffold was verified with the best angiogenic effect and osteogenic performance.

Due to the locally sequential release of BMP-2 and Sr ions, BMP-2/SrHA@PCG scaffold showed great improvement in the repair efficiency of bone defect, as evidenced by the micro-CT analysis and histological observation. In this study, BMP-2 was efficiently loaded onto the SrHA@PCG scaffold by the physical adsorption through a bridge of polyelectrolyte layer. After implantation, BMP-2 adhered on the surface of scaffold could elicit robust response of osteogenic effect [[57], [58], [59]]. In the process of bone regeneration, angiogenesis was coupled with bone formation, where BMP-2 was demonstrated to promote angiogenesis through the activation of PI3K/Akt, MEK/ERK, and Id-1/integrin α6 signaling cascades [60]. Additionally, it found that BMP-2 could regulate macrophages to stimulate the expression of angiogenic factors and osteogenic differentiation of bone marrow stromal cells, thus accelerating the angiogenesis and osteogenesis by manipulating the osteoimmune environment [44,61]. In order to further improve the angiogenesis and osteogenesis, simultaneous delivery of BMP-2 and other angiogenic factors was readily designed, such as vascular endothelial growth factor (VEGF) and stromal cell-derived factor 1 (SDF-1) [62,63]. As for the Sr-incorporated scaffolds, Sr ions released form the scaffold significantly promote bone regeneration. Importantly, Sr-doped biomaterials facilitated the expression of angiogenesis genes by activating the ERK/p38 signaling pathway *in vitro* and modulated macrophage phenotypes to promote early angiogenesis *in vivo* [8,64,65]. As a result, the scaffold containing BMP-2 and Sr ions showed the ability to achieve better vessel formation and bone regeneration. Here, benefiting from the porous structure and polyelectrolyte modification, the nanofibrous scaffold not only obtained improved loading efficiency of bioactive factor, but also allowed rapid and massive tissue ingrowth. And what is more, BMP-2 and Sr ions were assembled and released in a controlled manner to evoke a synergistic effect on bone regeneration. Therefore, in our design, a hierarchically structured scaffold was employed to incorporate SrHA nanoparticles that can accurately mimic ECM structures and chemical composition to natural bone. Furthermore, the polyelectrolytes modification was adopted to endow the composite scaffold with excellent hydrophilicity and enhanced cell proliferation and tissue ingrowth. Importantly, spatiotemporal delivery of BMP-2 and Sr ions from the polyelectrolytes-modified scaffold was realized to achieve a synergistic effect on bone regeneration. As a result, a dual-factor delivery biomimetic scaffold which had enhanced tissue infiltration and bone regeneration abilities was developed for obtaining augmented outcome of bone repair. Taken together, the current systematic study revealed that our designed BMP-2/SrHA@PCG scaffold exhibited desirable osteogenic ability for accelerating the efficiency of bone repair.

## 4 Conclusion

In the present study, we designed a biomimetic PCG scaffold incorporated with BMP-2 and SrHA to realize the controlled release of BMP-2 and Sr ions. The resulting scaffold was characterized by highly macroporous and nanofibrous structure. Owing to the successful coating of chitosan/gelatin by layer-by-layer assembly, improved hydrophilicity and protein immobilization efficiency were achieved. Furthermore, the polyelectrolytes modified-scaffold showed improved cell proliferation, and significantly promoted the cellular infiltration and neotissue formation within the scaffold after subcutaneous implantation. The SrHA incorporation not only improved the compressive strength of scaffold, but also allowed the release of bioactive Sr ions. Accordingly, a sequential release of BMP-2 and Sr ions from the scaffold was documented, in which BMP-2 released rapidly in the initial time and followed by a long-term sustained release of Sr ions. With the controlled release of BMP-2 and Sr ions from the dual-factor loaded scaffold, a synergistic effect on the osteogenesis was demonstrated both *in vitro* and *in vivo*. In the rat calvarial defect model, the dual-factor delivery scaffold significantly facilitated the healing of bone defect. Therefore, our proposed biomimetic scaffold would be a promising dual-factor delivery system for the treatment of bone defect.

## Credit authorship contributions statement

**Xiaojun Zhou:** Investigation, Methodology, Data curation, Writing-original draft. **Zunjuan Wang:** Investigation, Validation. **Tao Li:** Validation, Methodology. **Zhonglong Liu:** Validation, Formal analysis. **Xin Sun:** Validation, Formal analysis. **Weizhong Wang:** Conceptualization, Investigation, Validation. **Liang Chen:** Conceptualization, Supervision, Writing-review & editing. **Chuanglong He:** Conceptualization, Supervision, Project administration, Funding acquisition, Writing-review & editing.

## Declaration of competing interest

The authors declare that they have no known competing financial interests or personal relationships that could have appeared to influence the work reported in this paper.

## Data Availability

Data will be made available on request.
