# Supplementary material for: Enhanced tissue infiltration and bone regeneration through spatiotemporal delivery of bioactive factors from polyelectrolytes modified biomimetic scaffold
Source: Mater Today Bio. 2023 May 24;20:100681. doi: 10.1016/j.mtbio.2023.100681 (PMC10250921; doi:10.1016/j.mtbio.2023.100681)
Supplement: Multimedia component 1 [file mmc1.docx]

**Supplementary Materials**

**Enhanced tissue infiltration and bone regeneration through spatiotemporal delivery of bioactive factors from polyelectrolytes modified biomimetic scaffold**

Xiaojun Zhou,^a^ Zunjuan Wang,^a^ Tao Li,^b^ Zhonglong Liu,^c^ Xin Sun,^b^ Weizhong Wang,^a,*^ Liang Chen,^d,*^ Chuanglong He^a,*^

^a^State Key Laboratory for Modification of Chemical Fibers and Polymer Materials, Shanghai Engineering Research Center of Nano-Biomaterials and Regenerative Medicine, College of Biological Science and Medical Engineering, Donghua University, Shanghai 201620, China.

^b^Shanghai Key Laboratory of Orthopaedic Implants, Department of Orthopaedic Surgery, Shanghai Ninth People’s Hospital, Shanghai Jiao Tong University School of Medicine, Shanghai 200011, China.

^c^Department of Oral & Maxillofacial-Head Neck Oncology, Shanghai Ninth People’s Hospital, Shanghai Jiao Tong University School of Medicine, Shanghai 200011, China.

^d^Department of Joint Surgery, Zhongshan Hospital of Traditional Chinese Medicine Affiliated to Guangzhou University of Traditional Chinese Medicine, Zhongshan 528400, China.

^*^Corresponding authors: Tel: 86-21-67792666; Fax: 86-21-67792666

E-mail addresses: wz_wang@fudan.edu.cn (W. Wang); chenliang211@163.com (L. Chen); hcl@dhu.edu.cn (C. He).

**Supplementary Figures and Tables**





**Fig. S1** TEM image of SrHA.


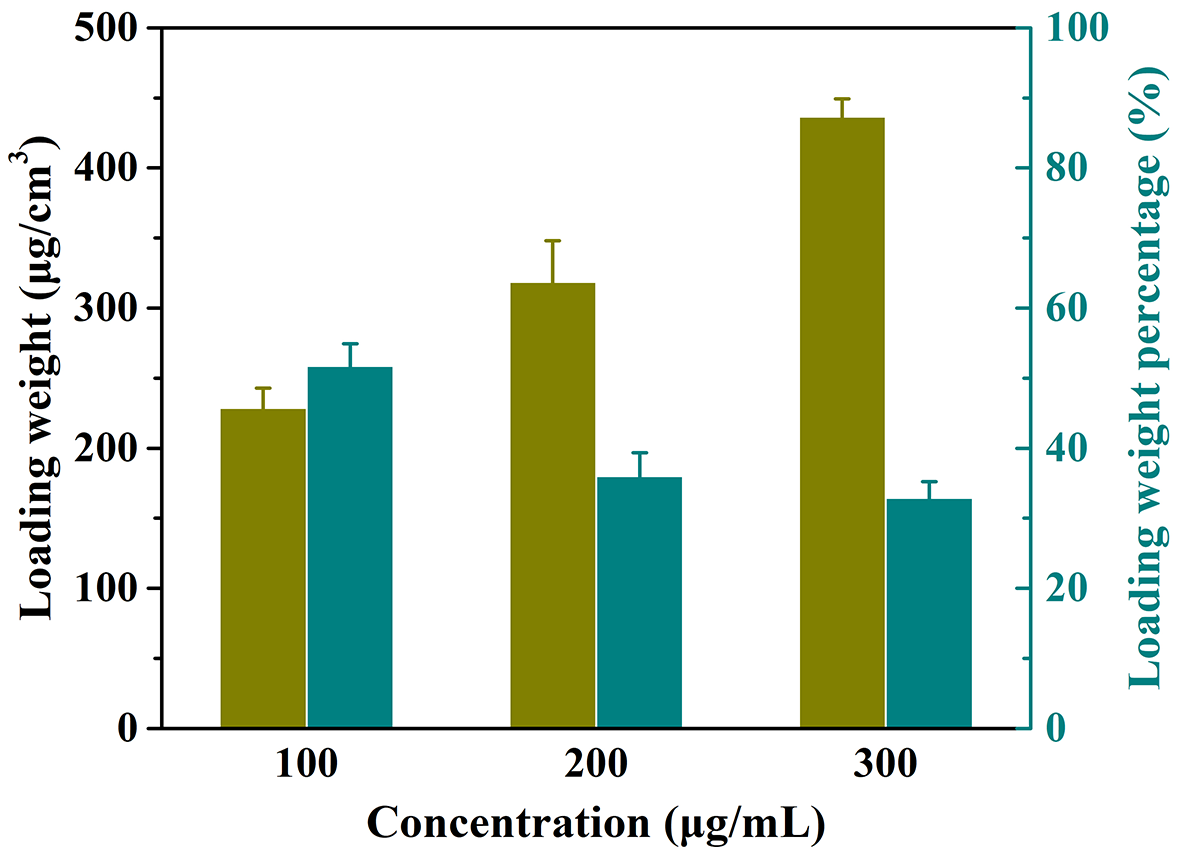


**Fig. S2** Protein binding efficiency of SrHA@PCG scaffold by soaking in different concentrations of BSA.


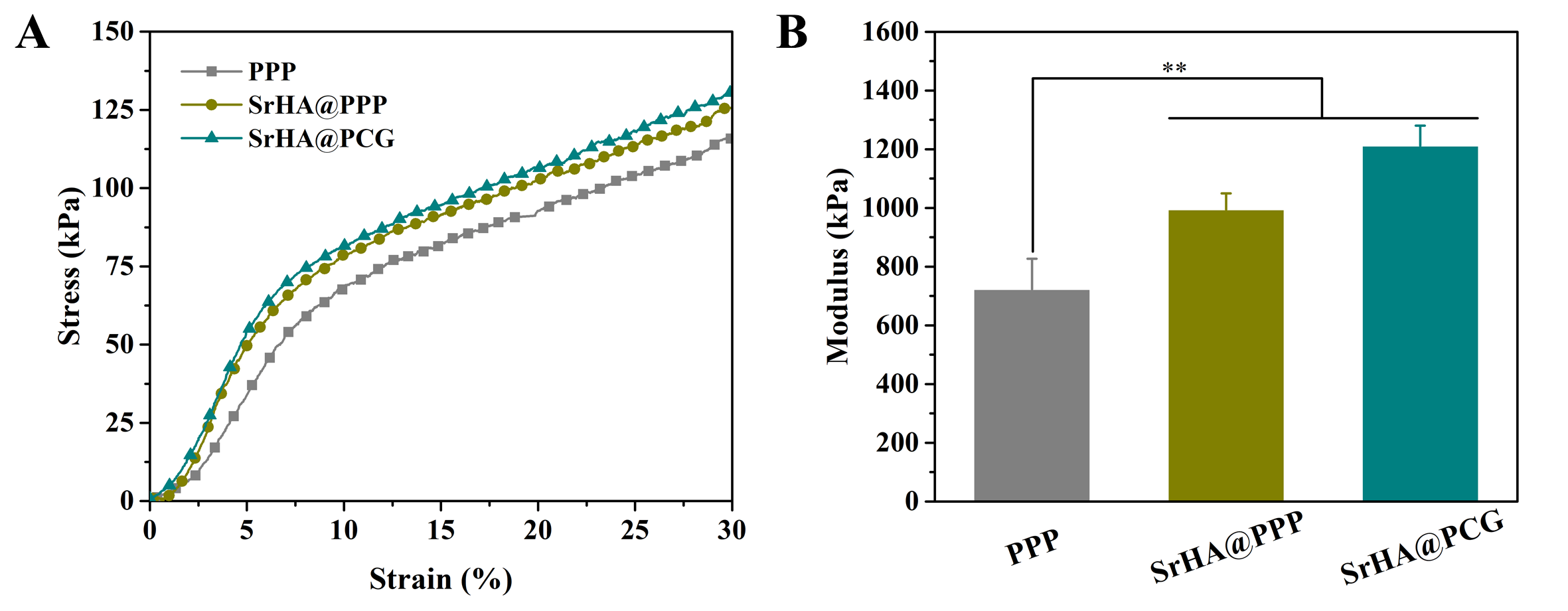


**Fig. S3** (A) Stress-strain curves of different scaffolds under wet state. (B) Compression modulus of different scaffolds under the wetted state.


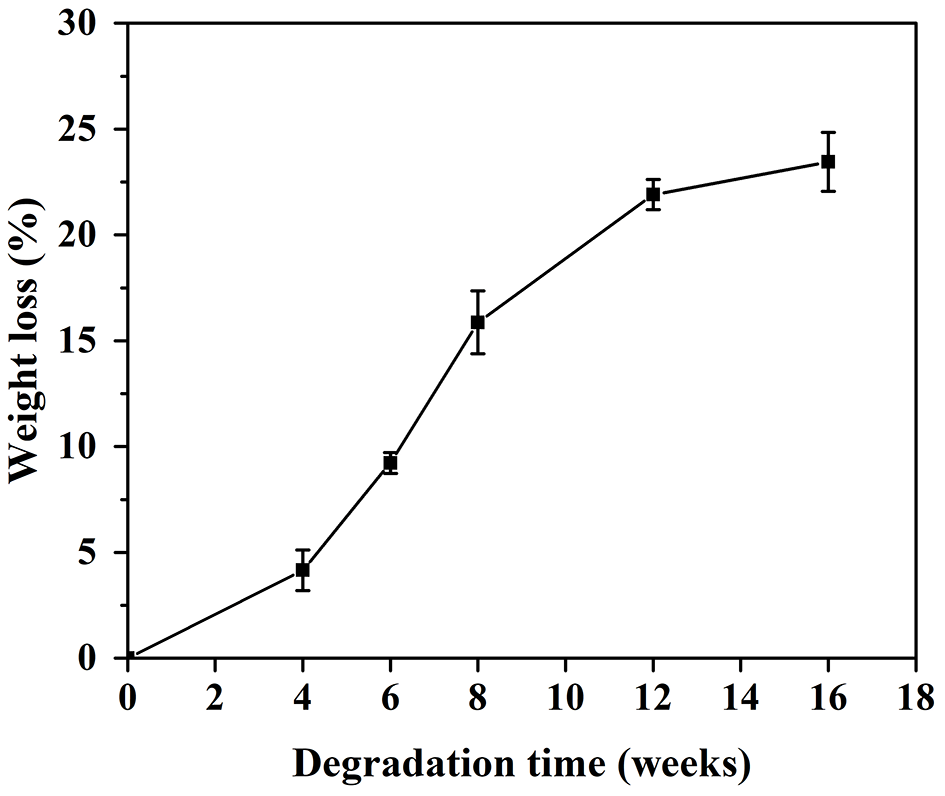


**Fig. S4** The degradation properties of SrHA@PCG scaffold over time.


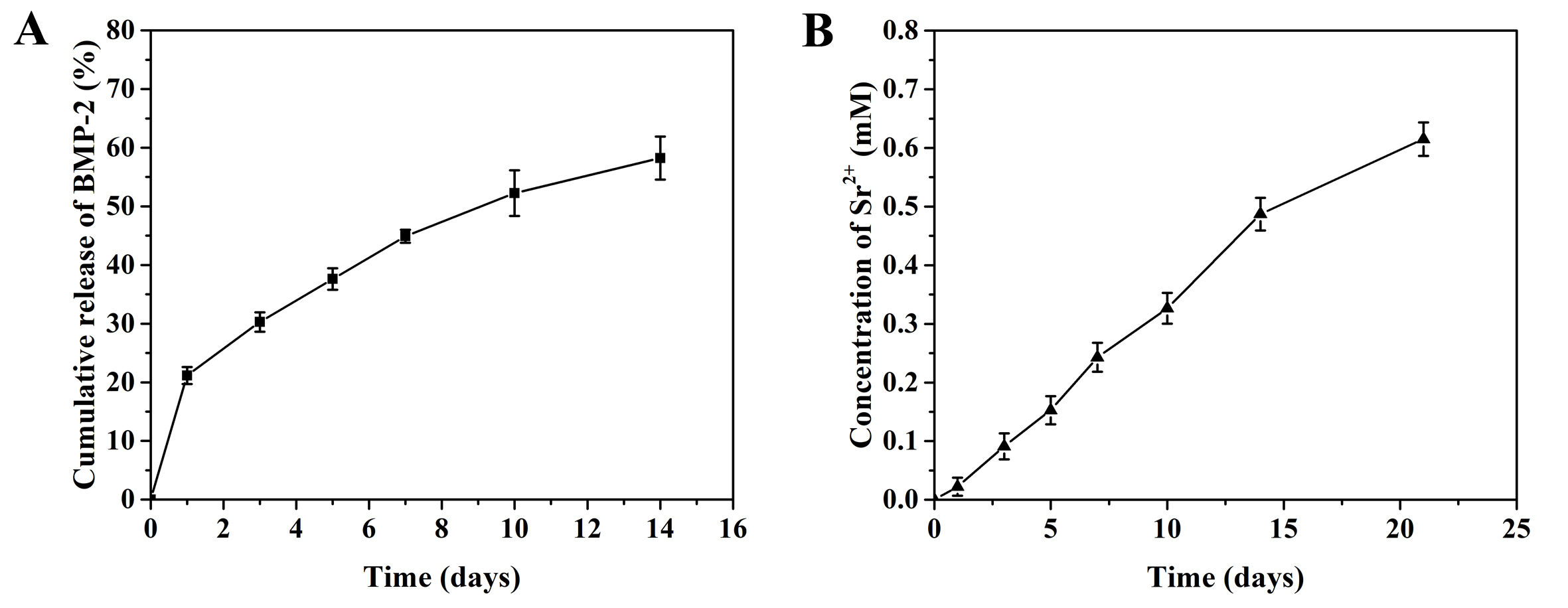


**Fig. S5** (A) *In vitro* release behavior of BMP-2 from BMP-2@PCG scaffold. (B) *In vitro* release behavior of Sr ions from SrHA@PCG scaffold.

**Table S1** Primers for qRT-PCR analysis in BMSCs.

| Gene | Primer sequences |
| --- | --- |
| Runx2 | Forward 5'- ACGTACCCAGGCGTATTTCA -3'  Reverse 5'- GCTGGATAGTGCATTCGTGG -3' |
| ALP | Forward 5'- CAAGGACCAACTACAACCA -3'  Reverse 5'- AGGGAAGGGTCAGTCAGGTT -3' |
| OPN | Forward 5'- AGCCATGAGTCAAGTCAGCT -3'  Reverse 5'- ACTCGCCTGACTGTCGATAG -3' |
| OCN | Forward 5'- AATAGACTCCGCGCTACCTC -3'  Reverse 5'- GCTAGCTCGTCACAATTGGG -3' |
| GAPDH | Forward 5'- CAAGTTCAACGGCACAGTCA -3'  Reverse 5'- CCCCATTTGATGTTAGCGGG -3' |
